# Neurological sequelae in survivors of cerebral malaria

**DOI:** 10.11604/pamj.2013.15.88.1897

**Published:** 2013-07-06

**Authors:** Isaac Oludare Oluwayemi, Biobele Jotham Brown, Olusola Adetunji Oyedeji, Margaret Adefiola Oluwayemi

**Affiliations:** 1 Department of Paediatrics, Ekiti State University Teaching Hospital, Ado-Ekiti, Nigeria; 2 Department of Paediatrics, College of Medicine, University of Ibadan/ University College Hospital, Ibadan; 3 Ladoke Akintola University Teaching Hospital, Osogbo, Nigeria; 4 Institute of Child Health, College of Medicine, University of Ibadan, Nigeria

**Keywords:** Cerebral malaria, neurological sequelae, children, Nigeria

## Abstract

**Introduction:**

Cerebral malaria is a common cause of neurological sequelae and death in childhood. Information on persistent neurological sequelae post hospital discharge and their predisposing factors are scarce.

**Methods:**

This is a prospective study describing persisting neurological impairments post discharge among children treated for cerebral malaria. In addition the study was designed to investigate the frequency of persistent neurologic deficits and the risk factors for their persistence in these patients. The case records of 160 patients treated for CM at the Paediatrics Department of University College Hospital, Ibadan from January 2004 to November 2006 were reviewed to recruit cases. Recruited survivors were then followed up for information concerning the presence and persistence of neurological sequelae.

**Results:**

A total of 160 children aged 9 months to 134 months were admitted and treated for CM during the study period. One hundred and thirty one (81.9%) survived while 29 (18.1%) died. The 131 survivors of cerebral malaria consisted of 64 boys and 67 girls. Neurological sequelae occurred in 13.7% of survivors of cerebral malaria at discharge and 4.6% at follow up. Six children with neurological deficits at discharge had persistence of deficits 6 months post-hospital discharge and one at 24 months. No associations were found between hypoglycemia, anemia, age, sex and multiplicity of convulsions, and persistence of neurologic sequelae. The persisting neurologic deficits among survivors at follow up were: memory impairment (1.5%), seizure disorders (0.8%), visual impairment (0.8%), speech impairment (0.8%), monoparesis (0.8%) and hyperactivity (0.8%) at follow up. The longest persisting sequelae lasted for at least 24 months.

**Conclusion:**

Neurologic deficits are not uncommon complications of CM. Neurologic sequelae may persist for as long as 24 months or more in survivors of childhood CM. There is no association between the risk factors for neurologic deficits and persistent neurologic sequelae.

## Introduction

*Plasmodium falciparum* is the most common cause of malaria worldwide [1]. It is responsible for almost all the mortality from malaria and affects the central nervous system causing neurological deficits and cognitive sequelae [1–3]. The social and economic burden of malaria in malaria-endemic countries is immense [4].

Cerebral malaria is the most severe neurological presentation of acute falciparum malaria, the clinical hallmark of which is the presence of coma [3]. It is a diffuse encephalopathy associated with seizures in at least 80% and status epilepticus, in up to a third of cases [5–7]. Multiple or prolonged seizures in cerebral malaria have been reported to be associated with epilepsy later in life [8–10]. The case fatality rate of cerebral malaria ranges between 5 and 50 percent [5]. Although most survivors make a full recovery, neurological sequelae such as hemiplegia, speech problems, cortical blindness and epilepsy occur in 3-31 percent [6, 8, 9]

Despite the growing recognition that *Plasmodium falciparum* malaria significantly threatens child survival and long-term impairment of cognition often follows early insult and injury to growing brains, inadequate attention has been given to the impact of malaria on general cognitive and behavioural development of children after discharge from the hospital by previous reports [9, 11]. Most of the previous studies report neurological sequelae in the acute phase or at discharge from the hospital in cerebral malaria [5, 12–14]. There are very few reports on the long term neurological sequelae in survivors of childhood cerebral malaria [8, 9, 11]. This study therefore focuses on describing persisting neurological impairments among children treated for cerebral malaria after a period of at least six months from the time of diagnosis. The study was carried out at the Paediatric Department of the University College Hospital, Ibadan, Nigeria and the homes of the patients in Ibadan community.

## Methods

The present study is a cross sectional study. The subjects were children who were previously admitted and treated for cerebral malaria at least six months before the time of study, who were then followed up for presence or persistence of neurological sequelae. Ethical clearance was obtained from The Joint University of Ibadan and University College Hospital Ibadan Ethical Committee. Informed verbal consent was obtained from the parents or guardians of the children and explanation was made to the children as much as possible to ensure their optimal cooperation.

All children with cerebral malaria admitted into Children Emergency ward between January 2004 and November 2006 were identified from admission records book and their case record files were retrieved from the Records Department of the University College Hospital. Baseline demographic and clinical data of patients at the time of diagnosis of cerebral malaria was extracted from the case notes using a structured case record form. The homes of affected patients were traced, using the contact addresses and mobile phone numbers in the case records. Enquiries were made to exclude other causes of brain injury between discharge and time of review. During the home visit, information was obtained from the caregivers. General clinical and detailed neurological examination (motor, gait, vision, speech, hearing, other cranial nerves, and intelligence quotient assessment) was carried out on the survivors of cerebral malaria and the information obtained was recorded in a structured case record form. Children with neurological deficits needing further evaluation were comprehensively reviewed at University College Hospital, Ibadan by specialists such as the ophthalmologist and physiotherapist in order to determine the exact deficit and its extent with the view of managing appropriately.

Data was analyzed using SPSS 11.0 for Windows (SPSS Inc., Chicago, USA). The patients were grouped based on age and the presence or absence of neurological deficit. Data was presented as frequency tables and mean ± standard deviations (x ± SD). Selected clinical and laboratory factors such as duration of coma, nutritional status, multiple seizures, anaemia and hypoglycemia were compared in those with and without sequelae. Analysis for significance was by use of Chi square or Fishers exact test, where counts were less than 5. P values less than 0.05 was regarded as significant.

Neurologic sequelae were defined as impairment of neurologic or cognitive function. Specifically they include: impairment or loss of function in musculoskeletal system, these includes paralysis or paresis manifesting as inability to walk, staggering, monoparesis, hemiparesis, quadriparesis; memory impairment (unusual forgetfulness of recent events), hyperactivity (excessive activity on the part of the child), seizure disorder (two or more seizures after discharge from hospital, unprovoked by fever), speech Impairment (difficulty in verbal expression), hearing impairment (inability to hear, or turn toward sound), and visual impairment (inability to visually follow brightly coloured objects or pick such objects with open eyes) [2].

## Results

### General characteristics of the study patients

A total of 160 patients aged 9 months to 134 months were admitted with diagnosis of cerebral malaria during the study period; 131 (81.9%) survived while 29 (18.1%) died. Of the 131 survivors of cerebral malaria, 18 (13.7%) had neurological deficits at discharge. Four of the 18 survivors with neurological deficits at discharge were lost to follow up in the community while 6 (4.6%) survivors had persisting neurological sequelae for more than 6 months after discharge from the hospital.

### Age and sex distribution

The 131 survivors were made up of 64 males and 67 females giving a male to female ratio (M:F) of 1:1.05. The age distribution of survivors shown in Table 1 reveals increasing frequency from infancy with a peak in the 36 - 47 months age group. The majority (71.8%) of children were under 5 years of age.

**Table 1:** Age distribution of survivors of cerebral malaria

Age groups (months)	Number of cases
6 – 11	4
12 – 23	19
24 – 35	25
36 – 47	30
46 – 59	17
60 – 71	17
72 – 83	4
84 – 95	5
96 – 107	5
108 – 119	2
120 – 131	2
132 – 143	1

### Duration of follow up and Frequency of neurological sequelae in survivors of CM

The survivors were followed up for a period that ranged from 6 months to 33 months with a median duration of 17.5 months from discharge to follow up. Eighteen (13.7 percent) of 131 survivors had neurological deficit(s) at discharge. Four of these were lost to follow up, giving an attrition rate of 22.2 percent among patients with neurologic deficit. Of the 131 survivors 6 had persisting neurological sequelae at 6 months, constituting 4.6 percent of all survivors and 33.3 percent of the 18 survivors with neurologic deficits at discharge. Thirty-eight neurologic deficits were identified among the 18 children at discharge. Of these thirty-eight deficits, 22 (57.9%) resolved completely while 9 (23.7%) persisted among 6 children. At follow-up, the most common persisting neurological deficit was memory impairment observed in 2 cases (Table 2). All the visual impairment resolved completely except a case later diagnosed as mild bilateral optic atrophy. The longest persisting neurologic sequela was a case of memory impairment lasting 24 months. Seven (18.4%) deficits in the 4 cases lost to follow up could not be reviewed (Table 3).

**Table 2:** Prevalence of neurologic deficits among survivors

Neurological Deficit	Deficit present at Discharge		Deficit at follow up	
	n	% of all survivors at discharge (N = 131)	n	% of all survivors (N = 131)
Visual Impairment	5	3.8	1	0.8
Speech Impairment	10	7.6	1	0.8
Memory Impairment	5	3.8	2	1.5
Seizure Disorder	1	0.8	1	0.8
Movement Disorder	9	6.9	-	0.0
Monoparesis	2	1.5	1	0.8
Quadriparesis	1	0.8	1	0.8
Hyperactivity	3	2.3	1	0.8
Hemiparesis	1	0.8	N/A	N/A
Hearing impairment	1	0.8	N/A	N/A

N/A = Not available (lost to follow up)

**Table 3:** Pattern of neurologic deficit in survivors of cerebral malaria

Age at Diagnosis (months)	Sex	Neurological Deficits		Duration between discharge &review (months)
		At Discharge	At Follow up	
60	F	Speech impairment, Movement disorder	Normal	24
60	M	Movement disorder, speech impairment, memory impairment	Normal	33
62	F	Right upper limb monoparesis, Speech impairment, memory impairment	Right upper limb monoparesis	12
22	F	Movement disorder	Normal	33
72	F	Movement disorder	Normal	26
84	F	Movement disorder, speech disorder, memory impairment	Normal	28
99	M	Right lower limb monoparesis	Normal	17
78	M	Memory impairment	Memory impairment	24
43	F	Hyperactivity	Normal	14
36	M	Visual impairment, speech impairment, movement disorder	Mild optic atrophy	6
46	M	Movement disorder, speech impairment	Normal	6
48	F	Hyperactivity, visual impairment, speech disorder, memory impairment	Memory impairment, hyperactivity	18
36	M	Visual impairment, hyperactivity, movement disorder	Hyperactivity, memory impairment	12
27	M	Seizure disorder, quadriparesis, speech impairment	Cerebral palsy with seizure disorder, spastic quadriparesis, speech impairment	8
47	M	Speech impairment, visual impairment, hearing impairment	Lost to follow up	
31	F	Hemiparesis	Lost to follow up	
40	F	Speech impairment	Lost to follow up	
11	M	Movement disorder, Visual impairment	Lost to follow up	

### Pattern of neurological sequelae in CM survivors

The 18 children with neurologic impairment at discharge had 38 neurologic deficits. The commonest neurological deficits at discharge were movement disorder and speech impairment in 9 children each, followed by visual impairment and memory impairment in 5 children each. Seizure disorder, hearing impairment, hemiparesis and quadriparesis occurred in one child each.

### Potential risk factors for neurological deficit in CM

No significant association was found between selected factors such as hypoglycaemia, severe anaemia, multiple seizures, under-nutrition, age, duration of coma among those with or without neurologic deficits at follow up. This result is shown in Table 4.

**Table 4:** Associations between selected factors and neurologic deficits at follow up

Selected factor	Neurologic deficit at follow up		X^2^	P
	Present	Absent		
**Hypoglycaemia (<2.2 mmol/L)**				
Yes	0	0	-	1.00
No	6	33		
**Anaemia (haematocrit ≤5g/dl)**				
Yes	0	2	0.38	0.71
No	6	31		
**Multiple seizures**				
Yes	5	15	3.75	0.06
No	1	18		
**Undernutrition**				
Yes	1	9	0.30	0.51
No	5	24		
**Age (<5years)**				
Yes	4	23	0.02	0.61
No	2	10		
**Duration of coma ≥ 2days**				
Yes	2	4	1.75	0.22
No	4	29		
**Sex**				
Male	4	14	1.20	0.39
Female	2	19		

## Discussion

Neurological impairment following CM is not uncommon and a number of studies have documented these [2, 6, 8–11, 15, 16]. The 13.7% neurological deficit prevalence in this study compares slightly lower to 17.7% and 15% documented by Bondi and Olumese et al respectively in the same centre some 30 and 25 years previously [8, 12]. The difference may be due to the different sample size and age groups of studied survivors. Also, the improvement in treatment modalities of CM over the past 25-30 years could explain the reduction in neurologic deficit estimates.

The prevalence of persisting neurological deficits in survivors of cerebral malaria in this study was 4.6%. This is an improvement over 9.7% prevalence reported by Bondi in the same centre 30 years earlier but higher than 2.6% prevalence obtained by Meremikwu et al in Calabar, Nigeria [8, 15]. The improvement in the prevalence of persisting neurological sequelae among survivors of cerebral malaria in the same centre over 3 decades could probably be due to improved case management of CM.

The persisting neurological deficits in this study were memory impairment, visual impairment, speech impairment, monoparesis, quadriparesis and hyperactivity. There was no persisting case of movement disorder which was the second commonest neurological deficit at discharge. Memory impairment was the most common persisting sequelae in this study. It usually becomes more obvious as survivors advance in age and face academic challenges. Our findings in the present study are similar to those of Cater et al at Kenya [16]. Subtle neuro-cognitive deficits, like memory impairment may be missed at discharge. This might find expression in later life when the survivors’ cognitive task increases in complexity [16]. Thus it is very important that survivors of cerebral malaria are well evaluated. Management should also be multidisciplinary with input from physiotherapists, special educationists, professionals or community rehabilitative workers, as the case may demand, in order to allow survivors to attain their maximum potential. This will also help protect these children from undue stress, neglect and abuse. Neurologically impaired survivors are prone to reduced opportunities in education, marriage, and employment.

The persisting neurological deficits found in this study, such as; ataxia, motor deficits, hearing impairment, visual impairment, speech difficulties, behavior difficulties, severe learning difficulties, seizure disorder, hemiplegia, spastic quadriplegia and cognitive impairment are very similar, with few exceptions, to those described in previous studies [2, 6, 8–11, 15, 16]. Visual impairment due to cortical blindness resulting from cerebral malaria usually resolve completely within 8 weeks of regaining consciousness [8, 13, 15, 17]. Diagnosis of the persisting case of visual impairment in this study as bilateral optic atrophy by the ophthalmologist underscores the need for multidisciplinary management.

The prevalence of seizure disorder in this study was 0.8%. This is higher than 0.2% reported by van Hensbroek et al in a study of 452 Gambian children who survived cerebral malaria [10]. It is however lower than the prevalence of 1.6% reported by Bondi thirty years earlier in the same center.8 Differences in clinical settings and geographical locations and improvement in the standard of care of CM may probably account for the variance in prevalence over time. Crawley et al in the study of 65 Kenyan children with cerebral malaria documented 1.5% prevalence of seizure disorder among survivors [7]. Different types of seizures including partial motor, generalized tonic'clonic and partial with secondary generalization were described during admission but post-cerebral malaria seizure disorders were poorly documented. The only persistent seizure disorder in this study was generalized tonic-clonic.

A third of the survivors who had neurological deficit at discharge in this study continued to have deficit at follow-up. This is comparable to 34.4% reported by Brewster et al in The Gambia and 38% in Kenya by Carter et al but lower than 54.5% reported by Bondi in the same centre 30 years ago [6, 8, 9]. The reduction in the proportion of survivors who continued to have neurological deficit at discharge in the same centre over the space of 30years has been previously attributed to improved standard of health and case management which is commendable. The difference could also be due to a longer duration between discharge and follow up assessment in this study (6 - 33 months) compared to 12 - 16 months in the earlier study by Bondi [8].

There was no statistically significant association between selected factors such as hypoglycaemia, severe anaemia, multiple seizures, under-nutrition, age, duration of coma and the presence or absence of neurologic deficits at follow up. These are at variance to the commonly reported findings in other studies [8, 12, 17]. This disparity may be due to a high index of suspicion and aggressive resuscitation of all unconscious children in our emergency room presently which might nullify the risks posed by such factors like anaemia, hypoglycaemia, prolonged coma and multiple seizures. A steady rise in the cases of CM survivors was recorded in the first four years of life. Most of these children were aged between 36 - 47 months. About three-quarters of the survivors of cerebral malaria during the 3 years study period were under 5 years of age. This is consistent with previous studies reporting that the most vulnerable age groups are the under-five children [7, 8, 14, 18].

## Conclusion

It is concluded that, the present study shows that neurological sequelae is common among survivors of CM. A minority have neurological deficits persisting beyond 6 months with memory impairment being the most common. The most vulnerable age groups were the under-five.
